# 2240 Shared decision making in child health: A qualitative study of parents of children with medical complexity

**DOI:** 10.1017/cts.2018.304

**Published:** 2018-11-21

**Authors:** Jody Lin, Catherine Clark, Bonnie Halpern-Felsher, Lee M. Sanders

**Affiliations:** Stanford University School of Medicine

## Abstract

OBJECTIVES/SPECIFIC AIMS: Children with medical complexity (CMC) comprise less than 5% of the pediatric population and over 40% of pediatric spending, yet receive poorer quality health care compared with other children. The American Academy of Pediatrics recently identified shared decision making (SDM) as a key quality indicator for CMC, but there is no consensus model for SDM in CMC. Objective: To create a model of SDM from perspectives of parents of CMC. METHODS/STUDY POPULATION: Interviews with parents of CMC explored SDM preferences and experiences. Eligible parents were ≥18 years old, English-speaking or Spanish-speaking, with a CMC <12 years old. Interviews were recorded, transcribed, and analyzed by 3 independent coders for shared themes using grounded theory. RESULTS/ANTICIPATED RESULTS: Interviews were with 31 parents [26 English speakers, median parent age 33 years (SD 11), median child age 3 years (SD 3.6)] in inpatient and outpatient settings. We identified specific, unique components of SDM that affect decision quality, the alignment of a decision with the parent’s preferences and values. Themes included: concerns about uncertainty of the child’s life trajectory, conflict during parent-provider communication, health system factors such as provider schedule; parent agency, and the influence of the source of information. DISCUSSION/SIGNIFICANCE OF IMPACT: Our findings provide specific components of SDM unique to CMC that can inform future research and interventions to support SDM for parents and providers of CMC.

